# Recognition of human action for scene understanding using world cup optimization and transfer learning approach

**DOI:** 10.7717/peerj-cs.1396

**Published:** 2023-05-23

**Authors:** Ranjini Surendran, Anitha J, Jude D. Hemanth

**Affiliations:** Department of ECE, Karunya Institute of Technology and Sciences, Coimbatore, India

**Keywords:** Deep learning, Convolutional neural network, Transfer learning, World cup optimization

## Abstract

Understanding human activities is one of the vital steps in visual scene recognition. Human daily activities include diverse scenes with multiple objects having complex interrelationships with each other. Representation of human activities finds application in areas such as surveillance, health care systems, entertainment, automated patient monitoring systems, and so on. Our work focuses on classifying scenes into different classes of human activities like waving hands, gardening, walking, running, *etc*. The dataset classes were pre-processed using the fuzzy color stacking technique. We adopted the transfer learning concept of pretrained deep CNN models. Our proposed methodology employs pretrained AlexNet, SqueezeNet, ResNet, and DenseNet for feature extraction. The adaptive World Cup Optimization (WCO) algorithm is used halfway to select the superior dominant features. Then, these dominant features are classified by the fully connected classifier layer of DenseNet 201. Evaluation of the performance matrices showed an accuracy of 94.7% with DenseNet as the feature extractor and WCO for feature selection compared to other models. Also, our proposed methodology proved to be superior to its counterpart without feature selection. Thus, we could improve the quality of the classification model by providing double filtering using the WCO feature selection process.

## Introduction

A scene is a collection of semantic information. A scene consists of multiple objects and complex relations between these objects. Identifying a scene visually is not a big challenge for human beings. But making a machine perform with human efficiency and understand a scene is a big task. Scenes (Greene & Oliva, 2009) may consist of indoor or outdoor images, aerial images, human activity, *etc*. Simply identifying the objects in a scene will not give complete information. It is also required to understand how these objects are related to each other. Understanding and classifying a scene (Walther et al., 2011; Vogel & Schiele, 2007; Cheng, Han & Lu, 2017) is still one of the most challenging tasks in the field of computer vision. Deep learning (LeCun, Bengio & Hinton, 2015) has found application in many areas of artificial intelligence (AI) research, including image classification (Szegedy et al., 2017), object detection (Girshick, 2015; Ren et al., 2016), human action recognition (Koppula & Saxena, 2016) image segmentation  (Badrinarayanan, Kendall & Cipolla, 2017; Chen et al., 2017), and so on.

One such category of scene understanding involves the recognition of human actions in a scene that involves different daily activities of humans that are related to cooking, drinking, waving hands, using mobile phones, *etc*. In the case of a visual scene of “drinking”, the detail we require is not simply detecting the person or the cup in the scene, but information about the activity taking place in the scene, namely, drinking. Thus, we need to categorize the scenes according to the semantic details involved. Human activity (Bux, Angelov & Habib, 2017) plays an important role in many real-world applications and has a wide scope of functionality in the areas of self-driving cars, robotics, entertainment, aviation, military applications, *etc*.

### Challenges and motivation

Human action recognition involves identifying various physical actions carried out by people that include different human poses, diverse objects, and a cluttered background. Based on the type of input data, human action recognition can be basically divided into three categories: sensor-data-based, video-action-based, and still-image-based activity recognition. Sensor-based and video-based methods are less challenging since multiple frames are available regarding an action, *i.e.,* more information is available regarding a subject’s motion. Therefore, the majority of research is happening in this field. Still, image-based action recognition, unlike the other two categories, is a more complex and challenging task due to a lack of spatial–temporal information. In this single frame, the action performed has to be identified.

In still images, the information regarding the actions needs to be extracted from the pose of the person, the surrounding multiple objects, or the distracting background within a single frame. The unavailability of a sufficient still image dataset is another major issue in the area of still image-based action recognition. Most state-of-the-art methods involve the use of data augmentation for increasing the amount of data, but this has the limitation of reducing the diversity of the data. Also, to improve the accuracy of the model, many researchers in this area use image annotation, which suffers from the disadvantage that it is time-consuming and costly. Owing to these several challenges, significantly less research has been done in the field of still image-based human action recognition. The aforementioned challenges motivated us to work in the area of human action recognition using still images.

### Contribution

There are different methods in which we can perform action recognition, and using pre-trained deep CNN models has improved the performance of classification tasks significantly. In our work, we have used the concept of transfer learning to train some of the most powerful CNN architectures on human action recognition for a still image dataset. We have used the existing pretrained models of AlexNet, SqueezeNet, ResNet101, and DenseNet201 for action recognition on the Stanford 40 dataset. The initial weights of these networks are set by the pre-trained weights obtained from the large ImageNet dataset. These networks are then trained on one of the benchmark human action datasets, which updates the network’s weights and biases on each layer for the classification task. This reduces the constraints in training these deep layer network architectures from scratch. In this work, we haven’t used any data augmentation or annotation techniques, as in many existing works. We have tried to improve the performance of our classification task by using feature selection based on the world cup optimization algorithm. Also, instead of using machine learning classifiers, we have used deep neural networks as the classifier. Our contributions to this work are as follows:

• We propose a transfer learning-based human action recognition model. Human activity images are first preprocessed using the fuzzy color stacking technique.
• Pre-trained dense layers of AlexNet-fc8, SqueezeNet-pool10, ResNet101-pool5, and DenseNet201-avg_pool were used for feature extraction in the experimental simulation.
• For improving the accuracy of the model, we have proposed the World Cup optimization algorithm for selecting the best features. This algorithm does not fall into local minima and provides the best solution globally.
• We have modified the classification layer of DenseNet to classify twenty-four human action classes.
• On a benchmark dataset, we show that our unique human action recognition method using a dense feature selection approach outstrips the current state-of-the-art methods.

The remaining sections of our article are organized as follows: ‘Related Works’ presents some of the related works; ‘Proposed Methodology’ details the proposed methodology, which includes datasets, preprocessing, feature extraction, selection, and classification; ‘Performance Matrix Evaluation’ deals with the evaluation of various performance matrices; ‘Experimental Results and Discussions’ shows the results of our experiment; ‘Comparison of Our Method with Different Existing Methods’ discusses the performance of our four pretrained models; and ‘Conclusion and Future Scope’ includes the concluding remarks.

## Related Works

Scene classification, one of the premier processes in the category of scene understanding, aims at analyzing a scene and categorizing it into a particular class. Traditionally, scene classification methods relied on pixel representation methods, then object-based methods, and later semantic-based methods. Pixels represent the basic information about any image. These pixel details were then utilized for classification. Due to issues regarding the resolution of the pixels and their size, later methodologies started adopting object-based methods for classification. Even though the object-based classification methods performed better than pixel-based methods, they suffered from a loss of semantic information. Thus, researchers started focusing on understanding the semantic features of a scene completely and developing better scene classification methods.

The classification process involving semantic details involved the use of characteristic features of the scene. The characteristic information included low-level, mid-level, and high-level features. The low-level features include color, texture (Ojala, Pietikainen & Maenpaa, 2002), shape (Oliva & Torralba, 2001), *etc*. These details of the image are extracted by the experts, and they are generated by the features extracted using locally generated handcrafted techniques like SIFT (scale invariant feature transformation) (Lowe, 2004), HOG (histogram of oriented gradients) (Dalal & Triggs, 2005), *etc*. These features were generated using manual methods and suffered from the problem of incomplete feature details. Classification models involving the use of middle-level features could solve the issues associated with low-level feature models. This is done by utilizing two steps. As the first step, mid-level features are abstracted by the use of traditional methods involving the Bag-of-Word (BoW) (Bahmanyar, Cui & Datcu, 2015) model approach. In this model, the images are considered a pool of “pictorial words”.

Other methods for feature extraction include spatial pyramid matching  (Lazebnik, Schmid & Ponce, 2006), Fisher vector coding (Zou et al., 2016), *etc*. In the second step, these handcrafted features are then fed to machine learning algorithms, which include k-means filters (Olshausen & Field, 1997), sparse coding (Abdi & Williams, 2010), principal component analysis (PCA) (Yao et al., 2011), *etc*. The efficiency of these methods depends on how talented the expert is at feeding the required features to the model. This has frequently limited the ability of traditional methods to imagine and represent the entire characteristic features of the image. Because the first structural methods developed for scene analysis focused on extracting details, they had difficulty generalizing to new, unseen scenes. The recent developments in the field of automation with powerful training-based learning models could elucidate the issues faced by the traditional methods of scene classification.

With the development of deep learning (LeCun, Bengio & Hinton, 2015), the concept of extracting high-level features for classification models paved the way for a new era. The deep learning models learned the features of a scene automatically from the large dataset they were trained with. The lower layers of the network learned the low-level features, whereas the top layers learned the high-level features of the scene. The convolutional neural network (CNN) (Fukushima, 1980; Lecun et al., 1989), which mimics the human brain, is one of the most popular and versatile deep learning models for classification applications. A convolutional neural network consists of an input layer, hidden layers, and output layers. The number of hidden layers indicates the depth of the deep network. The hidden layer is stuffed with a convolutional layer for extracting the different image features and is aided by a pooling layer for dimension reduction. The fully connected (FC) layer collects all of the features from the deeper layers.

Major applications in the field of deep learning happened with the development of the AlexNet (Krizhevsky, Sutskever & Hinton, 2012) deep learning model trained on the ImageNet (Deng et al., 2009) dataset. Many other deep CNN (Lecun et al., 1989) models elucidated the application of deep learning, including GoogleNet (Szegedy et al., 2015), VGGNet (Simonyan & Zisserman, 2015), SqueezeNet (Iandola et al., 2016), ResNet (He et al., 2016), DenseNet (Huang et al., 2017), and others. With the availability of large datasets and powerful processors, many research projects are being carried out with these deep CNN models in the area of scene classification. Some of the commonly used datasets for scene classification include Scene (Lazebnik, Schmid & Ponce, 2006), SUN (Xiao et al., 2010), Places (Zhou et al., 2014), NYU (Silberman et al., 2012), *etc*. Because of the extensive training and learning processes that use large datasets and powerful processors, these deep models have proven to be more accurate than traditional methods.

Transfer learning, a recent concept in deep learning, has improved many research works based on CNN models. One of the major limitations that affect the efficiency of deep learning models lies in the need for a large dataset and a powerful processor. A deep learning model can exhibit its maximum efficiency if it is fed a large dataset. One of the major drawbacks of using deep models is that there are only a few datasets available for some applications. In such cases, the concept of transfer learning can be applied to use deep models even with a limited dataset. The already-trained deep AlexNet (Krizhevsky, Sutskever & Hinton, 2012), GoogleNet (Szegedy et al., 2015), VGGNet  (Simonyan & Zisserman, 2015), SqueezeNet (Iandola et al., 2016), ResNet (He et al., 2016), and DenseNet (Huang et al., 2017) models of CNN  (Lecun et al., 1989) on the large dataset can be used to classify even small datasets. These models, whose weights are pretrained with the large dataset, can easily categorize the new classes of images with the knowledge they have acquired through pretraining. Pretrained CNN models learn the features from the dataset and extract millions of features automatically. These features are then classified by the classifier layer of the CNN model.

Feature selection is one of the techniques to improve the efficiency of the classification models. Among many feature selection methods, some recently developed nature-centered algorithms like the genetic algorithm (GA) (Mühlenbein, Schomisch & Born, 1991) and social-centered algorithms like the Particle Swarm Optimization (PSO) algorithm (Kennedy & Eberhart, 2001) have given solutions to the problem of optimization. These methods are used to select good features from the set of feature maps. The genetic algorithm is one of the simplest algorithms without any mathematical functions and has the ability to solve problems that have multiple solutions. However, it suffers from a drawback known as the “variant problem”, which arises due to the generation of corrupted blocks instead of actual blocks and thus generates random variables. The particle swarm algorithm is a faster and more intelligent method than the genetic algorithm. The success of this algorithm is due to the adaptation and involvement of individuals in the population. One of its major limitations is that it may get trapped in local minima.

A genetic algorithm was used in Desmet & Hoste (2018) for selecting parameters for the text classification. Sánchez-Illana et al. (2018) employed a particle swarm optimization algorithm for selecting the hyperparameters. In Kousias et al. (2018), the Bayesian optimization (BAO) algorithm is used for optimizing the parameters of the long short-term memory (LSTM) model. Stoean (2020) adopted a differential evaluation algorithm for tuning the layers of a convolutional neural network. In Badem et al. (2017), the artificial bee colony (ABC) algorithm is used for tuning the parameters of pre-trained CNNs for action recognition. Razmjooy, Sheykhahmad & Ghadimi (2018) used the world cup optimization algorithm to fine-tune the hyper-parameters for melanoma detection. Among the recent optimization algorithms that outperform other methods is the World Cup Optimization algorithm (WCO) (Razmjooy, Khalilpour & Ramezani, 2016), which was developed with inspiration from the FIFA competition. Unlike others, this algorithm does not get easily stuck in local minima. It has the advantage of having good convergence and will not generate random variables as its predecessors did. It also performs well in real-time applications.

Many state-of-the-art methods have used handcrafted methods and deep learning approaches for human action recognition. Most of the research in action recognition is video-based rather than based on still images. Traditional methods used handcrafted techniques to acquire the global description of the image. To recognize actions, Ikizler et al. (2008) used the oriental histogram, linear discriminant analysis (LDA), and SVM. The authors of  Delaitre, Laptev & Sivic (2010) have adopted a bag of features, part-based representation, and an SVM classifier to classify human actions. Yao & Fei-Fei (2010b) extracted the image features by using the grouplet technique and classified the human actions with an SVM classifier. In Yao & Fei-Fei (2010a), estimation of human poses and objects is modeled to classify the human-object interactions. Gupta, Kembhavi & Davis (2009) adopted the method of HOG to learn the interaction between humans and objects. Yao, Khosla & Fei-Fei (2011) combined random forests and decision trees in their work for categorization. Yao et al. (2011) adopted the use of object detectors based on attribute and parts of the action for understanding human actions. Olshausen & Field (1997) extracted the human-object interaction features by adopting weakly supervised learning with support vector machines.

With the success of convolutional neural networks (CNNs) in effectively extracting features from images, many researchers have used a variety of deep transfer learning models for performing human action recognition. Khan et al. (2015) adopted action-specific detectors based on transfer learning approaches to recognize the actions. They eliminated the burden of bounding box information during the test time, but the performance accuracy was limited to 75.5%. Zhang et al. (2016) employed CNN with multi-max pooling, Fisher vectors, and the GrabCut method to recognize the actions. They carried out the work with minimal annotation. It suffered from the issue that, for large numbers of images or classes in the dataset, the model may introduce heavy computation into the learning process. Yan, Smith & Zhang (2017) used the potential of fast R-CNN and a vector of locally aggregating descriptors (VLAD) coding on a spatial pyramid for the classification of human actions. They could identify both global and local spatial information simultaneously, but had the limitation of a large running time for feature extraction. They achieved an accuracy of 88.5%. Some of the existing works in human action recognition are covered in Table 1.

**Table 1:** Some existing works on human action recognition.

Study	Methodology used	Advantages	Disadvantages
Khan, Afzal & Lee (2022)	A hybrid CNN-LSTM model on Kinect V2 dataset with an accuracy of 90.89%	Performs better for frames with single person actions	Performance is affected when frame contains multiple people.
Muhammad et al. (2021)	Dialated CNN (DCNN) with attension mechanism using bi-direction long short-term memory (BiLSTM) on UVF11, UCF sports and J-HMDB datasets	Use of spatiotemporal information increased the performance	Single stream learning strategy was used on medium scale video based dataset
Yu et al. (2020)	Non-sequential CNN (NCNN) model having weight optimization and voting strategy model on Li’s and Willow action dataset	Use of Deep ensemble learning based has removed the burden of overfitting.	Model employed dataset with less background clutter.
Safaei & Foroosh (2019)	Transfer learning based fine-tuned spatial–temporal CNNs (STCNNs) using Saliency map and predicted optical flow methods.	Performance improved by extracting spatial- temporal information of still images.	Large annotated dataset was created to achieve the performance.
Saini et al. (2018)	A bidirectional LSTM-NN classifier and Microsodt Kinect	Showed efficientcy in extracting the domain specific features by using 3D trajectories from different body joints for video based action recognition.	Showed recognition rate of only 68.9%. More human poses and frames are needed for improving performance.
Sreela & Idicula (2018)	Features are extracted by Residual Neural Network (ResNet) and classified using SVM on Stanford40 and Pascal-VOC dataset.	Performance of action recognition improved by using high level CNN features and showed better performance for Pascal-VOC dataset (Precision-66.6%).	Model evaluated only 15 classes of human actions and performance was less for Stanford40 dataset .
Lavinia, Vo & Verma (2017)	Fused three deep CNN models,GoogLeNet, VGG-19 and ResNet-50 for feature extraction and SVM classifier. They used Stanford40 and People Playing Musical Instruments (PPMI) dataset.	Fusing multiple deep CNN models and deep ensemble learning, good performance was achieved, (Accuracy-81.14%)	Efficiency can be improved by fine tuning the model.

Many research works are focused on video-based action recognition due to the availability of spatial–temporal information regarding a human action. Research in the field of still image-based action recognition is comparatively lesser. So, we focused our work on still image-based action recognition. The limitations of existing works on human action recognition can be eliminated by using fine-tuned, deeply pretrained models. We do not need to train the pretrained models from scratch because they are already trained on a large dataset. By applying the knowledge gained from the large dataset, these pretrained models can learn the features of a smaller dataset and reduce the problem of overfitting. In our proposed work, we have used four pretrained models—the AlexNet, SqueezeNet, ResNet101, and DenseNet201—on the Stanford 40 action dataset. We have chosen ResNet and DenseNet pretrained models because these nets can overcome the vanishing gradient problem seen in many deep models.

The majority of action recognition models have shown better performance on medium and simple datasets. So, we have used the Stanford 40 dataset, which consists of a wide range of human actions with cluttered backgrounds, and accurately identifying these actions is a challenging task. As with many of the state-of-the-art methods, we have not used any data annotation or augmentation to improve the accuracy. Many research works on human action recognition include deep pretrained models as black boxes, and all the extracted features are used for classification. Irrelevant features can affect the accuracy of the model. The removal of these irrelevant features and the selection of superior features can improve the model’s accuracy. For feature selection, we have adopted the World Cup Optimization (WCO) algorithm because of its optimal feature selection properties compared to other optimization algorithms. The use of WCO algorithm is new in the field of human-object detection. Also, we have used a deep model classifier instead of a machine learning classifier. Our proposed model outperformed other models on human action recognition.

### Proposed methodology

**Figure 1:**
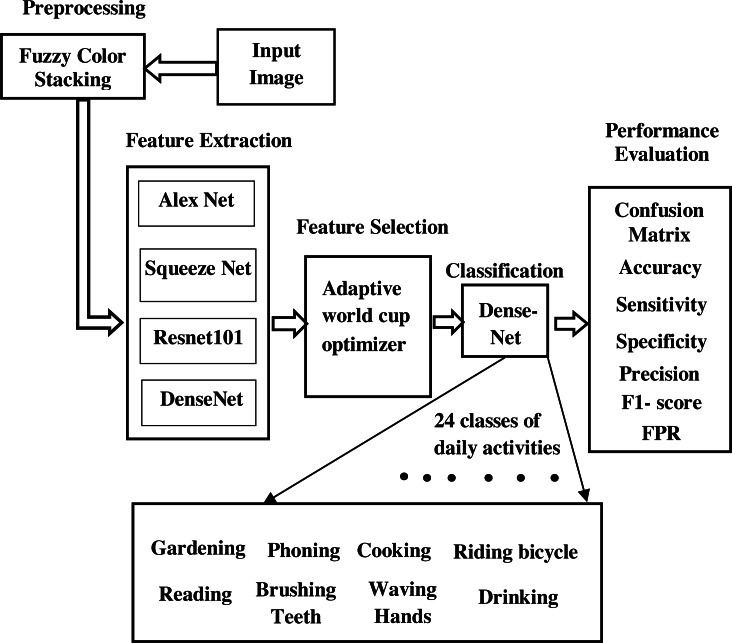
Block diagram of proposed model.

**Figure 2:**
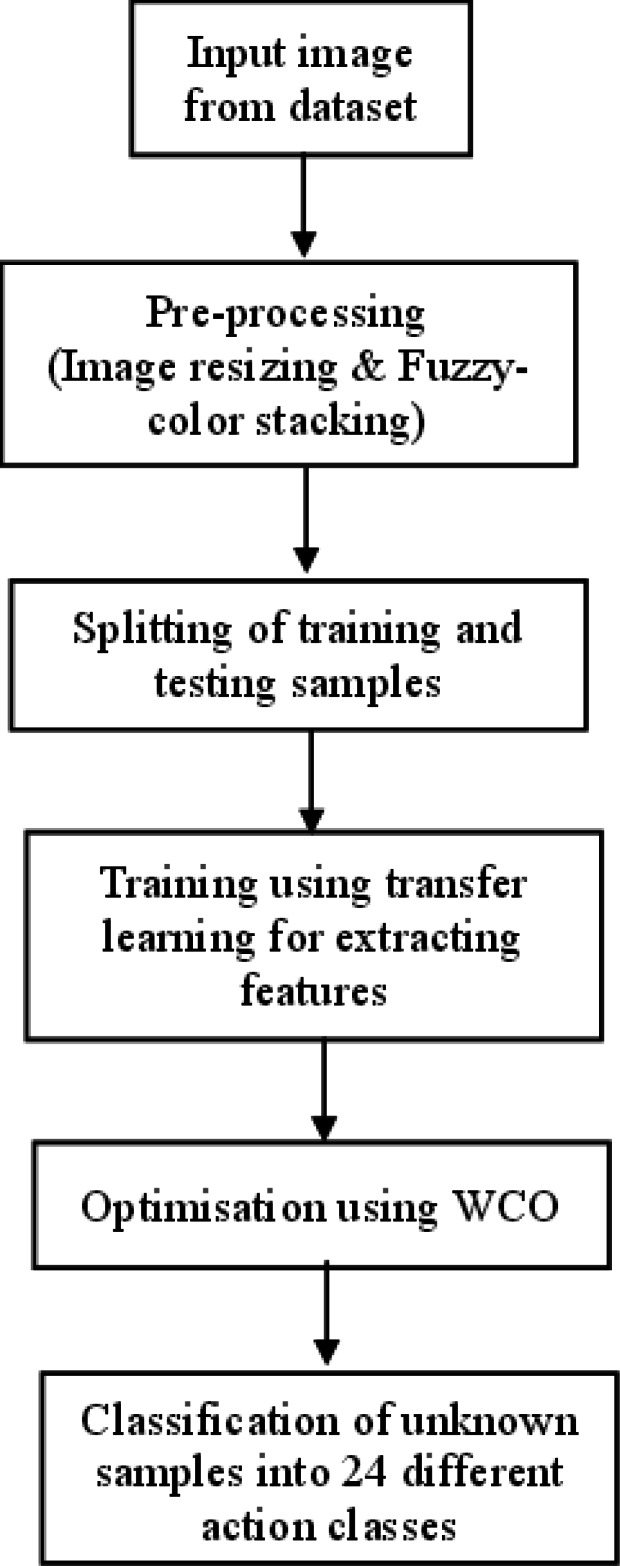
Proposed flow diagram of human action recognition.

The proposed work for the identification of the objects and to understand the human activities in a scene is shown in Fig. 1. Here we have adopted the transfer learning approach of the convolutional neural network (CNN) (Lecun et al., 1989) to annotate the scene. Instead of using a pretrained deep model as a black box that learns and performs classification, we aim at filtering the features learned by the deep model, selecting the best features from them, and then feeding the selected features to the classifier part. It worked on human-object interaction scenes and classified them into different daily activity classes. In this model, we have carried out our work on a standard data set: the Stanford daily activity dataset (Yao et al., 2011). Figure 2 shows the flow diagram of our proposed human action recognition model. In the first step, input images are pre-processed using the fuzzy color stacking technique to remove noise. The pre-processed images are then fed to the pretrained CNN model. The image features are extracted by the deep CNN model. In our work, the pretrained models used are AlexNet (Krizhevsky, Sutskever & Hinton, 2012), SqueezeNet (Iandola et al., 2016), ResNet101  (He et al., 2016), and DenseNet201 (Huang et al., 2017). After feature extraction, we have carried out a feature selection process where the most relevant features are selected from the generated feature map, and only these predominating features are then fed to the classifier. Feature selection is done using the adaptive World Cup optimizer technique. The classifier is DenseNet’s (Huang et al., 2017) 201-dimensional CNN pretrained model. It classifies the input image into 24 different classes of human activity.

### Dataset and preprocessing

We have carried out our proposed work on the Stanford 40-action dataset  (Yao et al., 2011; http://vision.stanford.edu/Datasets/40actions.html). This dataset includes various daily activities of humans in a scene. It is a collection of forty different actions performed by humans. Each category of the class consists of 180–300 action images, for a total of 9,532 scenes that relate to daily human activities. It showcases different human-object interaction scenes. We have collected 5,755 images with a total size of 565 MB. These 5,755 images were sorted and grouped into 24 classes of human actions. The number of images in classes varies from at least 182 to at most 315. Different classes of actions that we have used in our work include: applauding, blowing bubbles, brushing teeth, cleaning the floor, cooking, cutting vegetables, drinking, gardening, holding an umbrella, jumping, phoning, playing guitar, playing violin, reading, riding a bicycle, running, taking photos, texting a message, using a computer, washing dishes, watching TV, waving hands, writing on a board, and writing on a book. The number of image samples of each class used in our dataset and their size are shown in Table 2. As the first step, the different classes of image dataset are pre-processed using the stacking method of the fuzzy color technique to reduce the noise and filter the images. These stacked images are then fed to the feature extractor module.

**Table 2:** Database—Input Image.

Class	Number of images	Size (MB)
Applaud	284	25.7
Blowing bubbles	259	26.4
Brushing teeth	200	16.9
Cleaning the floor	212	15.9
Cooking	288	32
Cutting vegetables	188	17.1
Drinking	256	23.3
Gardening	199	26.2
Holding an umbrella	292	32.3
Jumping	295	30.1
Phoning	259	22.5
Playing guitar	289	28.7
Playing violin	260	26.9
Reading	245	25.4
Riding a bicycle	315	38.4
Running	251	26.4
Taking photos	197	19.9
Texting a message	193	17.4
Using a computer	229	21.7
Washing dishes	182	16
Watching TV	223	17.6
Waving hands	210	20
Writing on a board	183	15.2
Writing on a book	246	22.6
Total	5755	565

### Feature extraction using pre-trained models

The image features are extracted using the transfer learning technique. Pretrained deep CNN architecture models are used here to extract the different features of the image. As we know, the pretrained models are already trained with the existing large image dataset, so it becomes an easy task for these models to get trained with even a small dataset. With this knowledge transfer ability, these pretrained CNN models learn the image features with great accuracy. Here we have extracted the image features using the pretrained models: AlexNet (Krizhevsky, Sutskever & Hinton, 2012), SqueezeNet  (Iandola et al., 2016), ResNet101 (He et al., 2016), and DenseNet201 (Huang et al., 2017).

**Figure 3:**
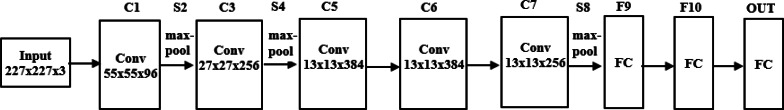
Architecture of AlexNet.

AlexNet (Krizhevsky, Sutskever & Hinton, 2012), a large deep convolutional neural network, was developed in 2012 to classify the one million images in an ImageNet (Deng et al., 2009) challenge into thousands of different classes. The architectural principle emphasized in the AlexNet model has remained a foundation for the CNN networks that are currently operational. The architecture shown in Fig. 3 can be viewed as a deeper and much larger network that consists of eight hidden weight layers, five convolutional layers used as feature extractors, and three fully connected layers used as a classifier. The first convolutional layer filters the input image using 96 kernels of size 11 × 11 with a stride of 3. The second convolutional layer filters the output of the first layer with 256 kernals of size 5 × 5. Between the first and second convolutional layers, there is maxpooling with stride 2 and size 3 × 3, and then a normalization is applied. The third, fourth, and fifth convolutional layers are connected to one another without any intervening pooling or normalization layers. The third convolutional layer has 384 kernals of size 3 × 3, the fourth convolutional layer has 384 kernals of size 3 × 3, and the fifth layer has 256 kernals of size 3 × 3. The fully connected layers have 4,096 neurons each, with ReLu in all layers.

**Figure 4:**
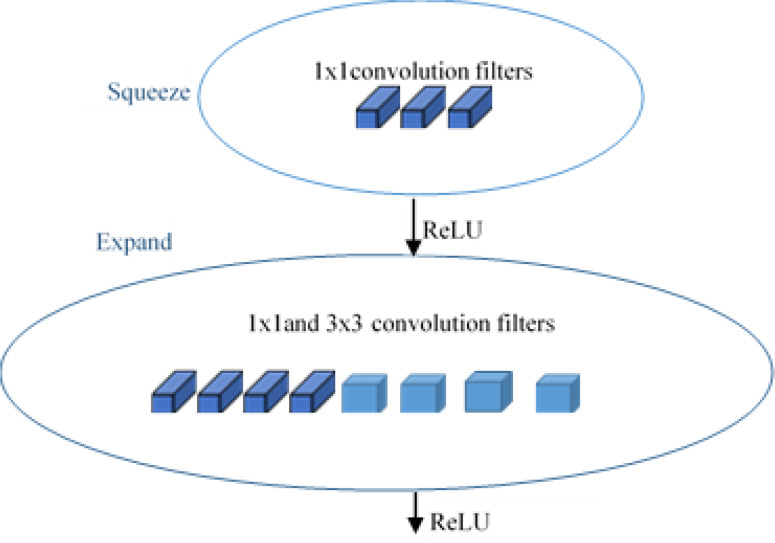
Fire module of SqueezeNet.

**Figure 5:**
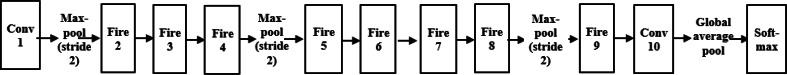
Architecture of SqueezeNet.

SqueezeNet (Iandola et al., 2016) is a smart and small convolutional neural network architecture that has the accuracy level of AlexNet (Krizhevsky, Sutskever & Hinton, 2012) on the ImageNet (Deng et al., 2009) dataset with parameters 50 times fewer and 3 times faster. SqueezeNet is designed with three special high-level strategy designs: 1 × 1 filters replace the 3 × 3 filters and form smaller networks, reducing the number of input channels to 3 × 3 filters, and providing a late downsampling in the network to achieve large activation maps in the convolution layers. The Fire Module, shown in Fig. 4, is the key idea introduced in the squeezenet module for achieving these designs. SqueezeNet carries out delayed downsampling by using max-pooling with stride 2 after the first convolution (conv1), the fourth and eighth fire modules (fire4 and fire8), and the tenth convolution (conv10) units. They squeeze the features with the squeeze layer consisting of 1 × 1 convolutional layers and then expand with a combination of 1 × 1 and 3 × 3 convolutional layers. Thus, the Fire Module shown in Fig. 5 is made up of two sections: one section has a squeeze layer that consists of 1 × 1 convolutional layers, and another section has an expand layer that has a combination of 1 × 1 and 3 × 3 filters. The squeeze layer consists of three convolutional layers of size 1 × 1, and the expand layer is a combination of four convolutional filters of size 1 × 1 and four 3 × 3 each.

**Figure 6:**
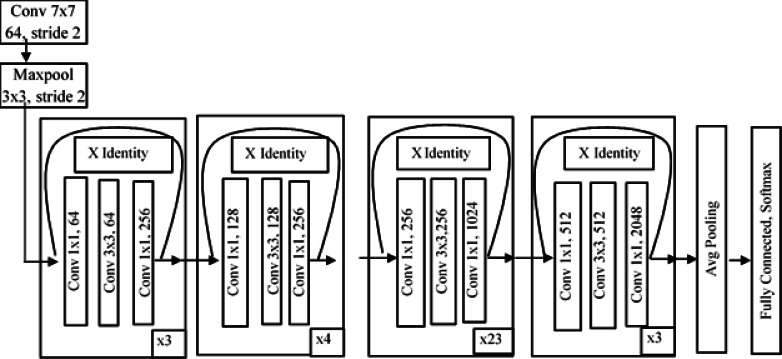
Architecture of ResNet101.

ResNet (He et al., 2016) is a residual deep neural network that handles the vanishing gradient problem. Residual networks use the concept of skip connections to resolve the problem of vanishing gradients. ResNet101 consists of 101 layers in its architecture. In ResNet, the convolutional layers are arranged like the traditional layers, but the original input is also added to the convolutional block output. This type of connection shown in Fig. 6 is known as a “skip connection” and can eliminate the problem of vanishing gradients. In this type of skip connection method, a few connections are skipped, and the final value will not be small quantity. The main idea behind ResNet is to make input and output equal, which means the network learns from the difference between the input and output. In a traditional network, we train the algorithm based on the output, but in a residual network, the algorithm is trained on the basis of the function of the input.

**Figure 7:**
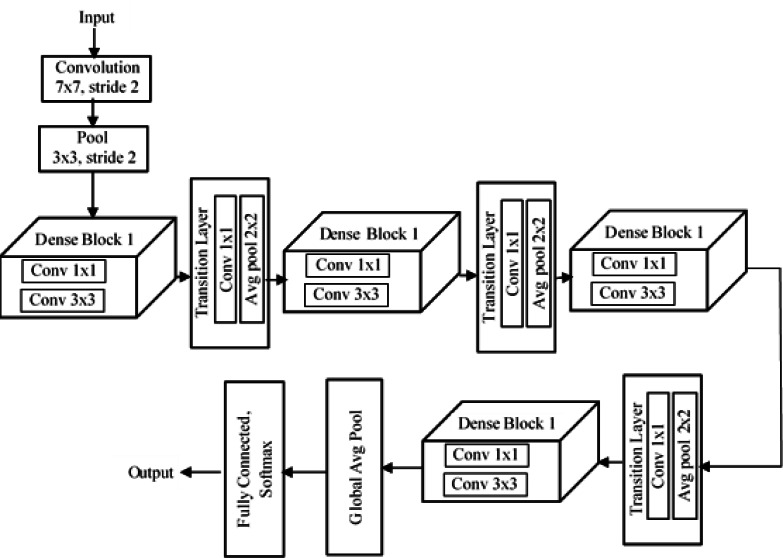
Architecture of DenseNet201.

DenseNet (Huang et al., 2017) is a dense convolutional neural network developed to improve the accuracy of a model by handling the problem of vanishing gradient. In deep networks, we have a lot of hidden layers between the input layer and output layer, and due to this long path, information vanishes before it reaches its destination. But DenseNet overcomes this vanishing gradient problem and offers high accuracy compared to other CNNs by simply connecting every layer directly to one another. Dense has different variants, among which is DenseNet201, which means there are 201 layers in the network. Input is given to a filter structure as shown in Fig. 7, whose size is 7 × 7 and uses a stride of 2. Then comes a pooling layer of filter size 3 × 3 and stride 2. The special structure found in DenNet is known as “dense blocks”. There are four dense blocks in the structure, and inside each block we have convolution layers of size 1 × 1 filter size and 3 × 3 filter size. The number of convolutional layers varies in each block.

Inside a dense block, each convolutional layer is connected to every other layer, and each layer receives the feature map from the previous layer. This helps each layer add some new features on top of the feature maps obtained in the previous stages. Thus, the new features get concatenated onto the existing feature map. We can concatenate the feature maps only if the size of the feature maps is the same. So inside each dense block, the size of the feature maps from each layer should be the same. For this reason, downsampling is not present in dense blocks. Downsampling is handled by another block in DenseNet, referred to as a “transition layer”. After every dense block, there is a transition layer. Each transition layer consists of a convolutional layer of size 1 × 1 and an average poling layer of size 2 × 2 and stride 2. The transition layer downsamples the feature maps obtained from the dense block. After the last dense block, there is a global average pool layer of size 7 × 7. Finally, there is a fully connected layer with a softmax function, and we get a classified output.

In our proposed work, we have used the pre-trained deep CNN models: AlexNet, SqueezeNet, ResNet101, and DenseNet201, for extracting the features from the preprocessed Stanford40 dataset. The different pretrained models and their layers that are selected for feature extraction are presented in Table 3. The input size that corresponds to the image input layer for AlexNet and SqueezeNet is 227 × 227 × 3, and for other models, the size is 224 × 224 × 3. AlexNet has three fully-connected (FC) layers, and the features are extracted from the *fc8* layer, which is the third FC layer of AlexNet. The *fc8* layer extracts 4,096 features per image for the 5,755 images in our dataset. SqueezeNet has 10 learnable layers, and the global average pooling layer, *pool10,* is selected to extract the features. For each of the 5,755 images in the dataset, the *pool10* layer extracts 1,000 features. ResNet101 is a widely used model and has 101 deep layers. The fully connected (FC) layer, *fc1000*, is selected for feature extraction. This layer extracts 2,048 features for each of the 5,755 images. DenseNet201 is a powerful deep model with 201 deep layers. The global average pooling layer, *avg_pool,* is selected for feature extraction. For each of the 5,755 images in the dataset, 1,920 features are extracted by the *avg_pool* layer.

**Table 3:** Feature extraction details of different models.

Model	Image input size	Feature extraction layer	Feature vector
AlexNet	227 × 227 × 3	fc8	5,755 × 4,096
SqueezeNet	227 × 227 × 3	pool10	5,755 × 1,000
ResNet101	224 × 224 × 3	fc1000	5,755 × 2,048
DenseNet201	224 × 224 × 3	avg_pool	5,755 × 1,920

### Feature selection using world cup optimization

The selection of features plays a vital role in the ability of a model to perform correct classification in the minimum amount of time. The pretrained deep CNN models get trained by the image dataset and generate a large feature map collection. These features include the details of an image that could be of less relevance. The majority of the extracted features may not be relevant for classification and may have an impact on the model’s accuracy. In order to solve this issue, a feature selection process can be used to select the best subclass from the original complete features. A general deep learning model classifies the images based on complete features learned by all the layers of the network, and the fully connected layer classifies these images. In our model, we have proposed a feature selection process before feeding these complete features learned by the network layer to the classification layer. Here, we used an adaptive world cup optimization algorithm (WCO) (Razmjooy, Khalilpour & Ramezani, 2016) to select relevant features from the feature bank. The existing search optimization algorithms suffered from the drawback that they fell into local minima. As inspired by the FIFA World Cup football competition, a new optimization algorithm was developed to optimize the performance of the mathematical functions. WCO is based on global heuristic search rather than the local minima search algorithm.

WCO algorithms work as follows: In this optimization algorithm, initial variable values are termed “team”, which forms clusters forming countries. After the teams have been formed, the continents’ scores are calculated. This is accomplished by calculating the mean and standard deviation for each continent, as shown below.

(1) \begin{eqnarray*}& & \text{Mean}(X)= \frac{1}{n} \sum _{i=1}^{n}Xi\end{eqnarray*}

(2) \begin{eqnarray*}& & \text{Standard Deviation}~(X),\sigma =\sqrt{ \frac{1}{n-1} \sum _{i=1}^{n} \left[ (Xi-\text{mean}(X))^{2} \right] ~}\end{eqnarray*}

(3) \begin{eqnarray*}& & \text{Rank}= \frac{(\beta \ast \sigma +\text{mean}(X))}{2} .\end{eqnarray*}

Where ‘*n*’ denotes the total number of members of continent ‘*X*’, ‘ *β*’ is the decrease or increase coefficient of ‘ *σ*’, and lies between [0, 1]. Based on the mean and standard deviation, each objective function is evaluated and ranked as shown below. With the ranks calculated, all the continents are sorted into two groups, one with good records and the other with poor records. The competition is then evaluated by first finding the teams with the lowest value on each continent. Later, the minimum among all the values is evaluated. If this evaluation is satisfied, then the process is terminated; otherwise, based on the top-value country record, the population is newly generated, and the process repeats till the challenge is completed. The World Cup optimizer model works by twice filtering the best solution. This kind of filtering is used for better feature selection but is not provided by other optimization algorithms. Rather than applying the complete features to the classifier model, we have given the most superior and relevant features for classification. This reduces the misleading errors, increases the accuracy of the model, and enhances its efficiency.

In our work, by using an adaptive world optimizer, we select the best features among all the input features obtained from the pre-trained model. The steps involved in feature selection that we have used in our work are summarised as follows:

• The input to the algorithm is the extracted features and its labels.
• By using the fitness function, the weight for each feature attribute is calculated.
• The fitness calculation and position update are carried out by setting the probability of individual learning at 0.85 and the probability of exploration learning at 0.1.
• The features that have a high fitness function (weight) are selected, and others are rejected.
• Finally, the index of the selected attributes is given as the output of the optimization algorithm.

In this work, the output of the pre-trained deep model, which are the features extracted from our training dataset, is considered for feature selection and separated into populations. Each population is provided with random samples of extracted image features. The most superior features are then selected by ordering the random features based on the generated cost function and fitness function. We have the features as the input and the corresponding labels as the target. For each column of features, we have the labels, and each particular feature is classified, with the probability of individual learning at 0.85 and the probability of exploration learning at 0.1, which predicts the label value. This predicted value is then compared with the input label values, and an error is calculated. Using this error value, we calculate the cost function, which is given as follows:

(4) \begin{eqnarray*}\text{Cost}=0.99\ast \text{error}+0.01\ast \frac{\text{number of selected features}}{\text{total number of features}} .\end{eqnarray*}

Here the fitness function is minimization of error, *i.e.,* the features having the lowest error value will be chosen and others will be discarded. For each population, we generate the cost function, and the fitness function is updated. Based on the updated fitness function, we update the randomly generated initial population. Each time the cost function is calculated, it is compared with the previous value. The one that has the minimum cost function is updated as the fitness value, and the others are discarded. This is repeated until the fitness function converges and the features with the highest fitness value are selected and others are rejected. This double filtering technique excels at world cup optimisation compared to other optimisation algorithms.

The feature selection process increases the performance of our model by removing irrelevant features. Each of our pre-trained models extracts more than 5,700,000 features from our dataset. The total number of features extracted by each model and their corresponding count of selected features using the world cup optimization algorithm are presented in Table 4. Comparing with the number of features extracted and selected by each feature extraction layer of the pre-trained models, AlexNet initially extracted 23,572,480 features that were reduced to 11,648,120 features upon feature selection. The feature selection reduced the 5,755,000 features extracted by SqueezeNet to 2,917,785 features. Initially, 11,786,240 features were extracted by ResNet101, and 5,881,610 of the best features were selected. DenseNet 201 initially extracted 11,049,600 features, and the 5,588,105 best features were selected by feature selection. These selected best features are then used for the classification into twenty-four classes using a modified deep learning model, DenseNet 201.

**Table 4:** Feature extraction and feature selection of models.

Model	Feature extraction layer	Number of features extracted	Number of features selected using WCO
AlexNet	fc8	23572480	11648120
SqueezeNet	pool10	575500	2917785
ResNet101	fc100	11786240	5881610
DenseNet201	avg_pool	11049600	5588105

### Feature classification using densenet

**Figure 8:**
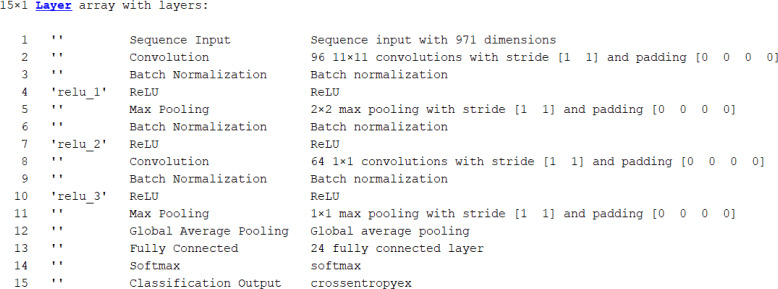
Layers of DenseNet201 as classifier.

The most relevant features selected by the World Cup Optimization algorithm (Razmjooy, Khalilpour & Ramezani, 2016) are fed to the classifier module. Here, the pretrained deep DenseNet network is used to classify the image. The fully connected layers perform the classification by using the superior features obtained from the feature selection module. For classifying our 24 classes, we modified the fully connected layers and pooling section. Rather than classifying based on all features, the network was designed to classify based on superior features obtained through World Cup optimisation. Here the input to the classifier is the selected features from the optimizer module. The dense network architecture that we have used as a classifier model in our research is shown in Fig. 8. The description of the network model is as follows: The input sequence layer accepts a size of 971. The network consists of convolutional filter layers of size 11 × 11 × 96, batch normalisation, and ReLU. This is followed by a 2 × 2 max pooling layer with a stride of [1 1], batch normalisation, and the ReLU layer. These layers are followed by the layer of a convolutional filter of size 1 × 1 × 64, batch normalisation, and the ReLU layer. The last layers are followed by a 1 × 1 max pooling layer with a stride [1 1], global average pooling, a fully connected layer for 24 classes, and a softmax layer with classification output.

**Figure 9:**
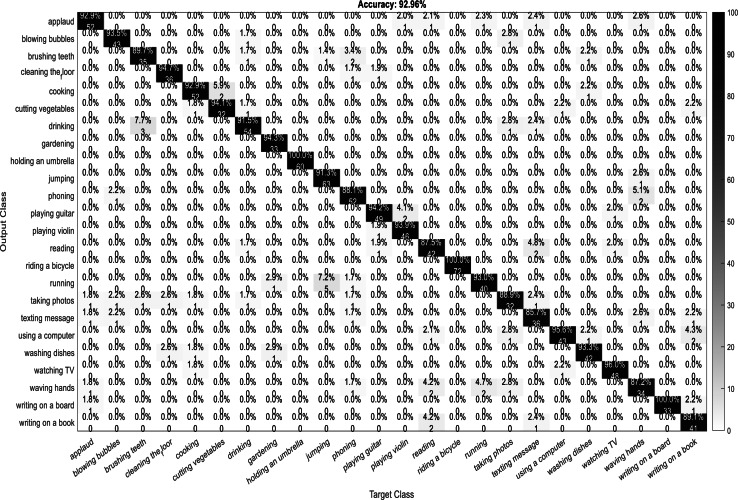
Fine-tuned classifier layer for DenseNet.

In our work, we have also modelled the feature extraction and classification by the DenseNet201 directly without using the feature selection module. The deep model densenet classifier has a default input size of 224 × 224, whose weights were already adapted to the ImageNet dataset and get updated with our image dataset. DenseNet201 has 201 layers with four dense blocks and three transition blocks. It has an initial 7 × 7 convolutional layer of 64 channels followed by batch normalisation, ReLU, and a 3 × 3 max pooling layer with stride 2. We removed the last three layers of the pre-trained model, *i.e.,* ‘fc1000’, ‘fc1000_softmax’, and ‘ClassificationLayer_fc1000’, and defined our new classification layers. These include a fully connected layer, a dropout layer, and a softmax layer. A fully connected layer is designed for 24 classes and is specified with a weight learn rate factor of 20 and bias learn rate factor of 20. We have used a 0.5 dropout factor for the dropout layer. Based on the modified classifier layers, our model classifies the images into twenty-four different daily activity classes. The fine-tuned DenseNet classifier layers are shown in Fig. 9. The classifier module with the feature selection module performed better than the model without the feature selection module.

## Performance Matrix Evaluation

The effectiveness of a deep learning model can be evaluated by analyzing its performance matrices. The quality or performance of a model is evaluated by using various matrices. These matrices are referred to as “performance matrices”. Evaluation of these matrices will provide information regarding the quality of the CNN model. In our work, we have calculated performance parameters like accuracy, sensitivity, selectivity, *etc*.

Accuracy is the simplest and most important measure of the performance of a network. It defines how many class predictions are correct out of the total count of predictions. Sensitivity defines as how many true or correct positive values are correctly predicted by the model, whereas specificity defines how many true or corrected false values are correctly specified by the model. These matrices are calculated using parameters like true positive (Tp), true negative (Tn), false positive (Fp), and false negative (Fn).

(5) \begin{eqnarray*}\text{Accuracy}& = \frac{\text{True positive}+\text{True negative}}{\text{True positive}+\text{False positive}+\text{True negative}+\text{False negative}} \end{eqnarray*}

(6) \begin{eqnarray*}\text{Sensitivity}& = \frac{\text{True positive}}{\text{True positive}+\text{False negative}} \end{eqnarray*}

(7) \begin{eqnarray*}\text{Specificity}~& = \frac{\text{True negative}}{\text{True negative}+\text{False positive}} .\end{eqnarray*}

We have also calculated various statistical parameters like precision, F1-score, and false-positive rate. These parameters give a measure of the effectiveness of the classifier.

(8) \begin{eqnarray*}\text{Precision}& = \frac{\text{True positive}}{\text{True positive}+\text{False positive}} \end{eqnarray*}

(9) \begin{eqnarray*}F1-\text{score}& =2\ast \frac{\text{Recall}\ast \text{Precision}}{\text{Recall}+\text{Precision}} \end{eqnarray*}

(10) \begin{eqnarray*}\text{False positive rate}& = \frac{\text{False positive}}{\text{False positive}+\text{True negative}} .\end{eqnarray*}

We have evaluated the parametric matrices for all four pretrained CNN models (AlexNet, SqueezeNet, ResNet101, and DenseNet201) used for feature extraction in our proposed work.

## Experimental Results and Discussions

We used the powerful tool MATLAB R2021a to conduct our overall experimentation. We have worked on an i7 Intel Core processor with an NVIDIA RTX GPU operating at a speed of 2.30 GHz and utilizing 16 GB of system RAM.

We tested the proposed model’s performance using the Stanford40 dataset, which contains images of various human activities that can be related to daily routines such as drinking, watching TV, gardening, running, and so on. The dataset is used to train different pretrained deep CNN models using the concept of transfer learning. Here we have used pretrained models of AlexNet, SqueezeNet, ResNet101, and DenseNet201 to extract the features. These features were then fed into a feature selection module, which employs a World Cup optimization algorithm to double filter the features before feeding the most dominant ones to DenseNet’s classifier.

Our dataset consists of a total of 5,755 images divided into 24 classes of actions. We have used 80% of our dataset to train the network and the remaining 20% to test our network models. Thus, we have 4,604 images for training and 1,151 images for testing. Out of the 4,604 training images, 20% are used for validation, with the remaining used for training. In order to train our proposed model, we use the ‘Adam’ optimizer with a learning rate of ‘*0.01*’. The gradient threshold is set to ‘*1*’ and the learning rate drops by a factor of ‘0.2’. We have completed the training process in 100 epochs with a mini batch size of 128.

**Figure 10:**
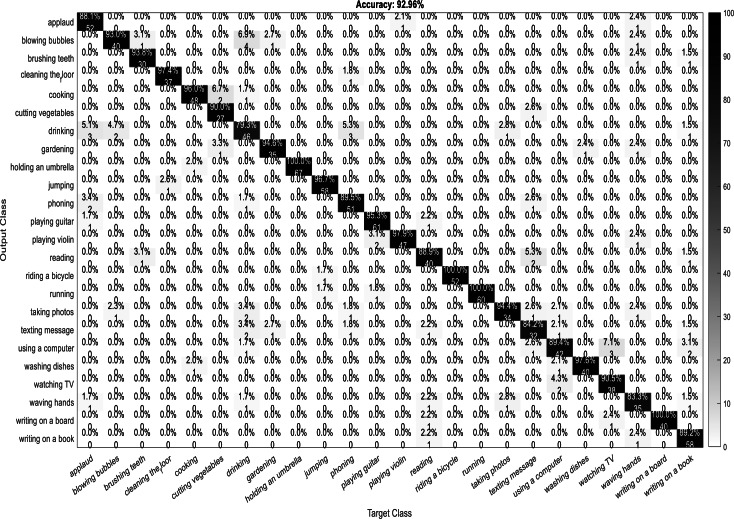
Confusion Matrix of DenseNet-DenseNet model.

**Figure 11:**
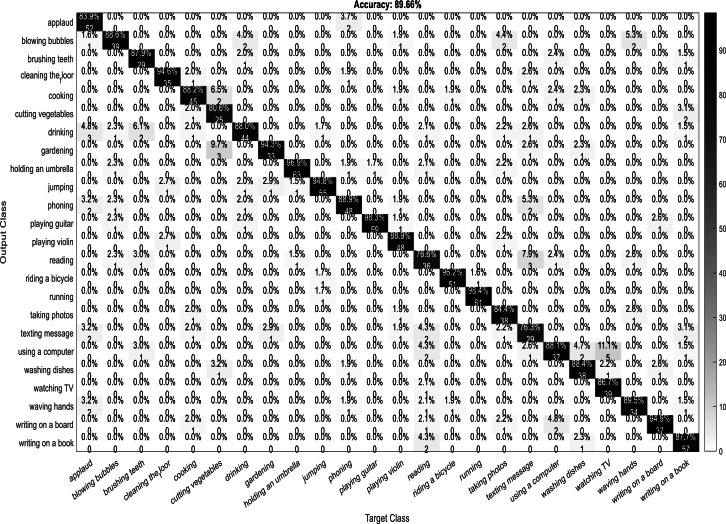
Confusion Matrix of ResNet-DenseNet model.

**Figure 12:**
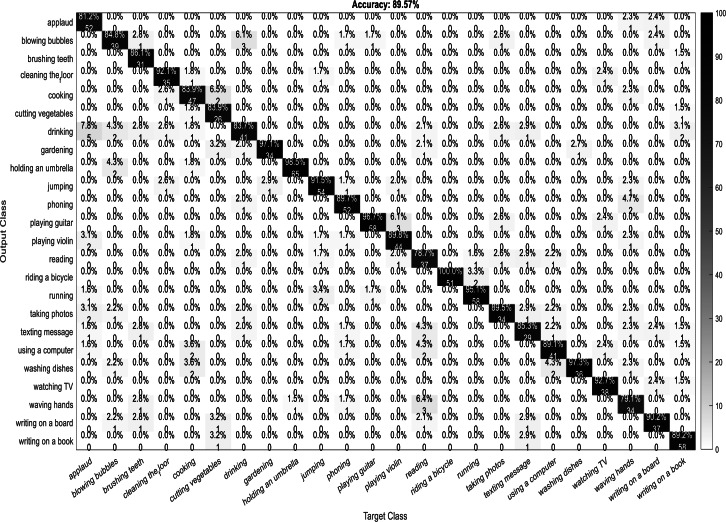
Confusion Matrix of AlexNet-DenseNet model.

**Figure 13:**
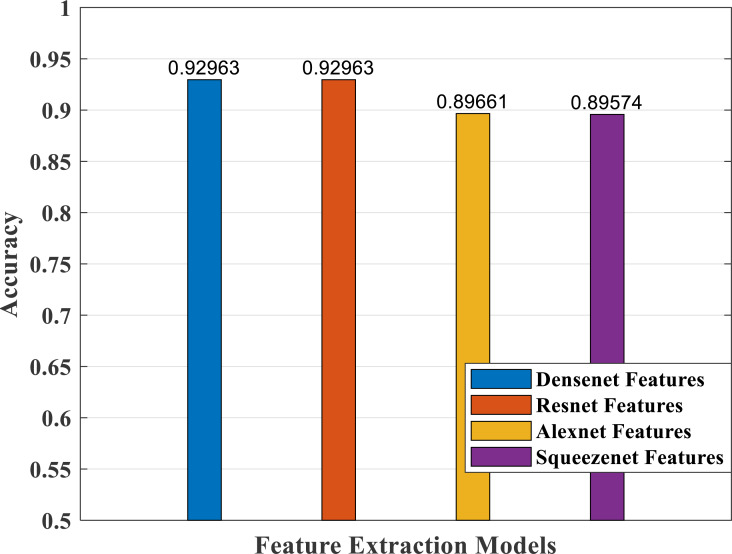
Confusion matrix of SqueezeNet-DenseNet model.

A confusion matrix, also known as an error matrix, is a two-dimensional tabular representation, with the actual class as the row elements and the predicted class as the column elements. For each class, it summarizes the count of predictions that are correctly and incorrectly classified. Thus, for a given set of data, the performance of a classifier can be evaluated using a confusion matrix. A confusion matrix is calculated for the different training models to analyze the different performance parameters of the classifier. Figs. 10, 11, 12 and 13 show the confusion matrix plots of the DenseNet-DenseNet model, the ResNet-DenseNet model, the AlexNet-DenseNet model, and the SqueezeNet-DenseNet model, respectively.

### Performance analysis—feature extraction, selection and classification

**Figure 14:**
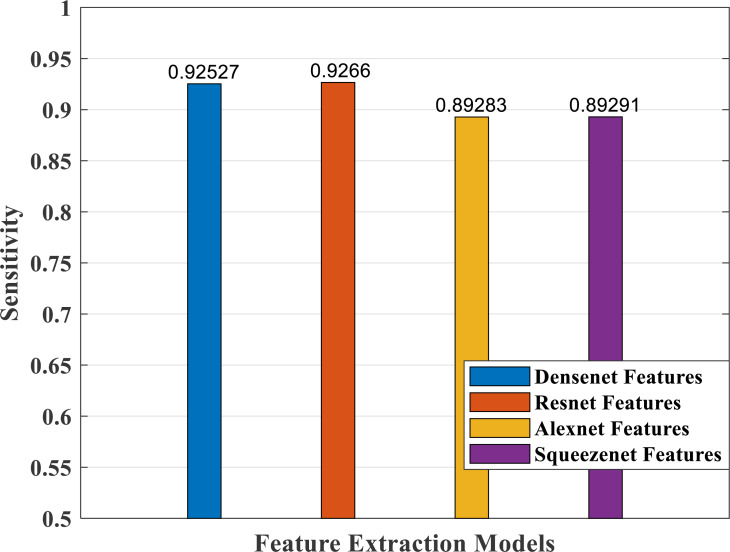
Accuracy measure plot of different CNN models.

**Figure 15:**
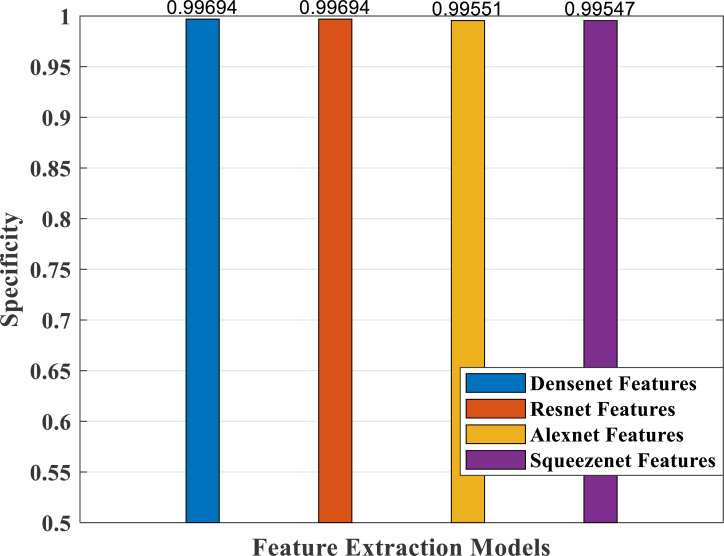
Sensitivity measure plot of different CNN models.

**Figure 16:**
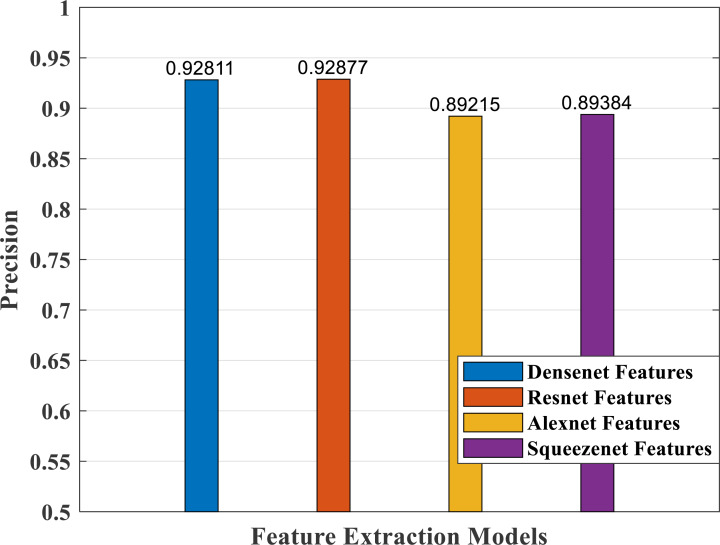
Specificity measure plot of different CNN models.

**Figure 17:**
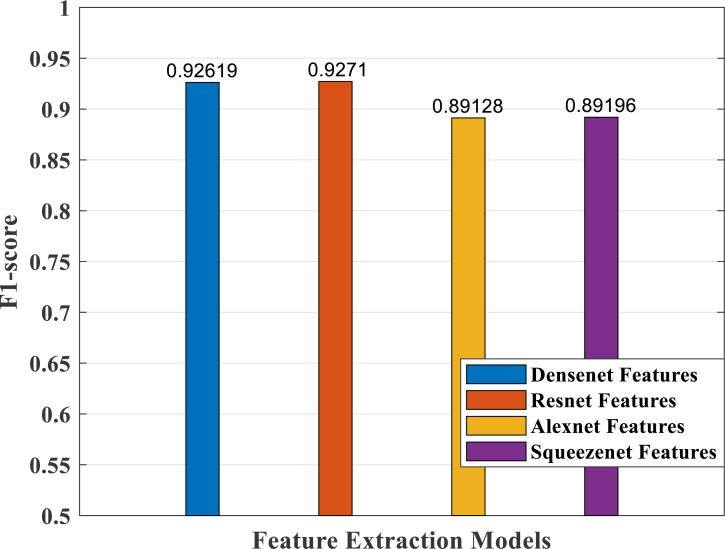
Precision plot of different CNN models.

**Figure 18:**
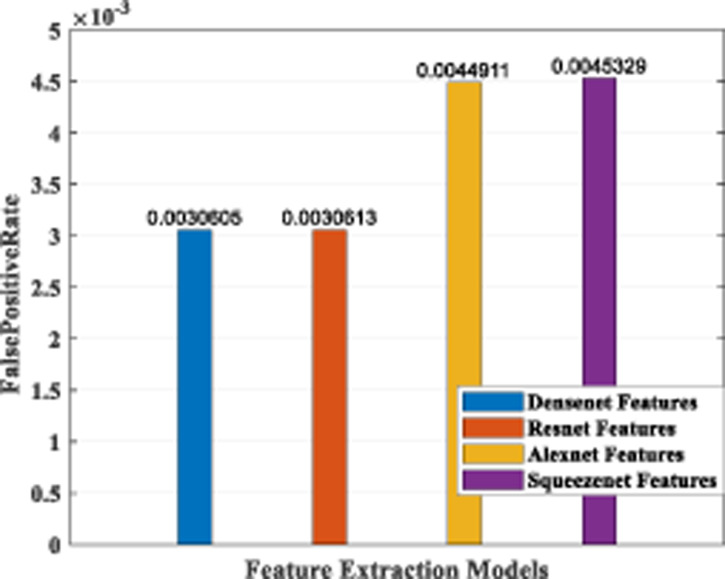
F1-score plot of different CNN models.

**Figure 19:**
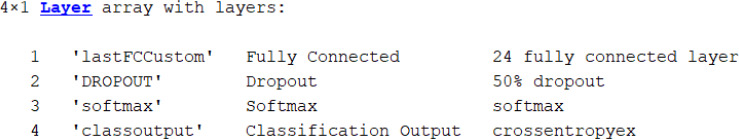
Plot of FPR for different CNN models.

In our proposed work, we have adopted the pretrained deep learning models to train our pre-processed Stanford40 dataset, which contains 24 classes of daily human activities. The pretrained AlexNet, SqueezeNet, ResNet101, and DenseNet201 architecture models extract the features from the image. The world cup optimizer algorithm was used to filter and select the most superior and relevant features from the feature map, which were then fed to the DenseNet classifier layer. Figs. 14, 15, 16, 17, 18 and 19 show the performance of different feature selection CNN models.

The accuracy plot of different CNN models is shown in Fig. 14 with comparable accuracy for DenseNet and ResNet compared with AlexNet and SqueezeNet models.

Figure 15 depicts the sensitivity measure analysis of the four models. It shows the best response for the densenet and resnet models and the lowest for the SqueezeNet model.

Figure 16 depicts specificity, which is a measure of correctly predicting the wrong classes. It shows a comparable good response for all the models.

The precision measure of a classifier model indicates how many actual predictions made are correct out of the total count of predictions. Figure 17 shows the precision values of the different models, with better response for both the DenseNet and ResNet models.

According to F1-score values plotted in Fig. 18, the DenseNet and ResNet models performed better than the AlexNet and SqueezeNet models.

The false-positive rate was also calculated for the four architecture models, and the results are shown in Fig. 19. It indicates that the DenseNet model and the ResNet model have a comparable low FPR, while AlexNet and SqueezeNet have a higher value.

**Figure 20:**
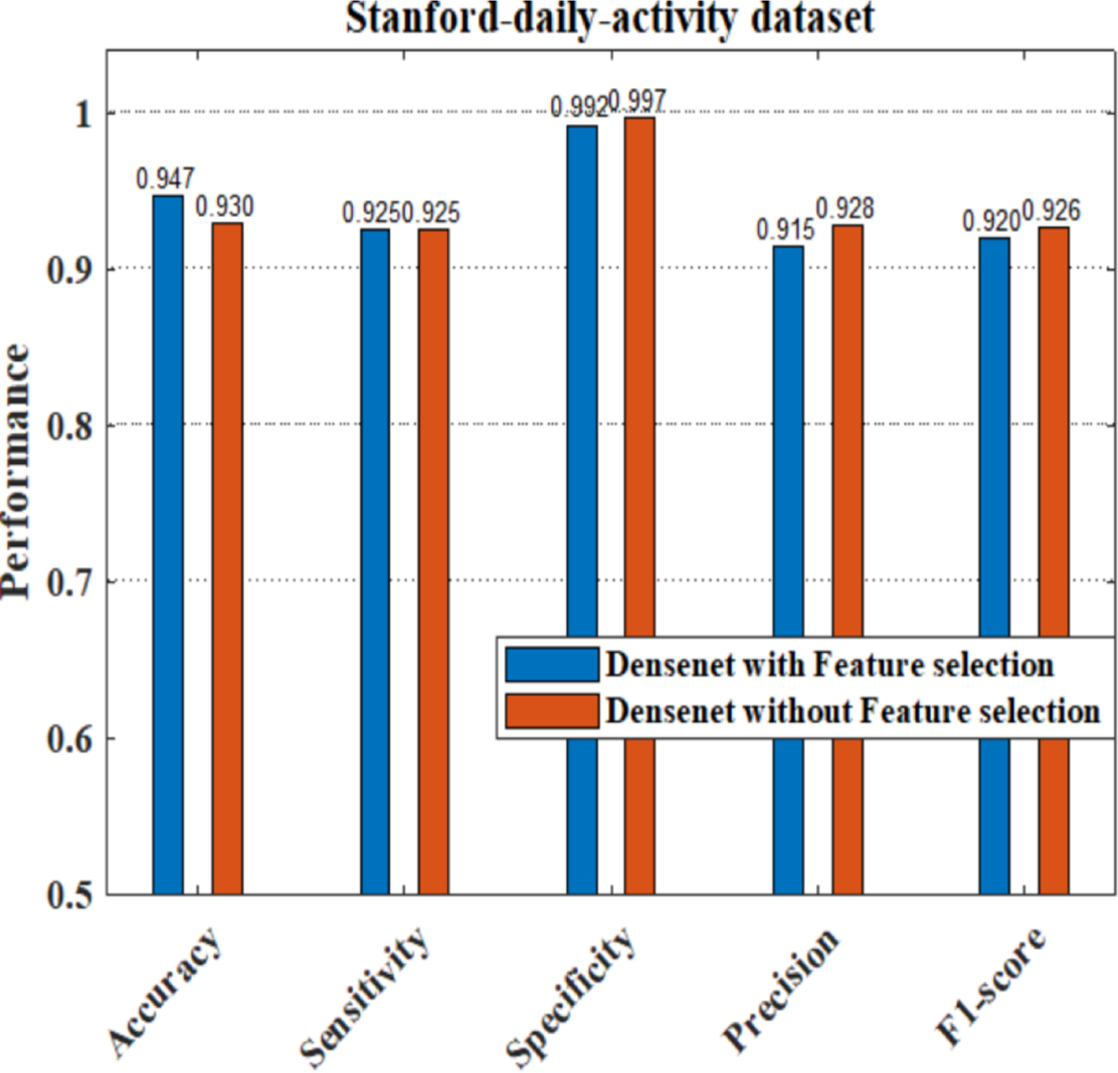
Comparison of DenseNet with and without feature selection.

The performance analysis of the proposed model having DenseNet201 and ResNet101 as the feature extractors shows a better response compared to other pretrained feature extractors. Here we have also applied the dataset to train the pretrained DenseNet201 deep model without feature selection, where the image features are learned by the deep layers of the DenseNet and the images are classified into 24 different classes. We have evaluated the performance of the DenseNet classifier with and without using the feature selection process. Figure 20 shows that our proposed model with a feature selection process shows a good response for the system parameters like accuracy, sensitivity, precision, and F1-score compared to the ordinary densenet model without feature selection.

## Discussions

As part of scene understanding, we proposed a model that can annotate a scene in order to analyze a scene with objects involved and identify the relationship between the objects. Here we have carried out the experiment with the Stanford daily activity dataset, which consists of 24 different classes of daily human activities. We have trained the pretrained models of AlexNet, SqueezeNet, ResNet101, and DenseNet201 for feature extraction. The world cup optimization algorithm is then used to identify the dominant or superior features among the entire feature map during the feature selection process. These superior features were then fed to the DenseNet classifier. The different pretrained deep learning CNN models with DenseNet as the classifier are evaluated for their performance matrices. The values of different performance matrices such as sensitivity, specificity, precision, F1-score, FPR, and accuracy for the various pre-trained models are shown in Tables 5, 6, 7 and 8. Our classifier model with DenseNet201 and ResNet101 for the classification of 24 scene classes gave an accuracy of 92.96% in comparison to the accuracy of AlexNet of 89.66% and SqueezeNet of 89.57%. The model also outperformed in the performance measures with a sensitivity value of 92.5%, specificity of 99.6%, precision of 92.8%, F1-score of 92.6%, and FPR of 0.0030 as compared to other models. From the analysis, by considering the number of features extracted by each model and evaluating their performance matrices, we conclude that for our given pre-processed input dataset, the DenseNet201 pre-trained model provides the best accuracy compared to other models. Table 9 compares the classification model’s different performance matrices with and without using the World Cup optimization method. The accuracy of the proposed model with feature selection is 94.7%, compared to the 93% accuracy value of the model without feature selection.

## Comparison of Our Method with Different Existing Methods

Many research studies are carried out using different machine learning and deep learning models to perform human action recognition. In this section, we have compared our research results with some recent research studies on still image action recognition. Here, we have performed the comparison for the research works on the Stanford 40 dataset. Even though our comparison finding may not be really reasonable since many research papers use different datasets, it provides a visualisation of the different classification approaches and their results. Table 10 shows the accuracy comparison of our action recognition model with other existing models for the Stanford 40 dataset.

**Table 5:** Performance matrices of AlexNet model for human action recognition.

Model	Sensitivity (%)	Specificity (%)	Precision (%)	F1-score	FPR
AlexNet—DenseNet201	89.28	99.55	89.21	89.12	0.00449
Accuracy (%)	89.66				

**Table 6:** Performance matrices of SqueezeNet model for human action recognition.

Model	Sensitivity	Specificity	Precision	F1-score	FPR
SqueezeNet—DenseNet201	89.29	99.54	89.38	89.19	0.00453
Accuracy (%)	89.57				

**Table 7:** Performance matrices of ResNet101 model for human action recognition.

Model	Sensitivity	Specificity	Precision	F1-score	FPR
ResNet101—DenseNet201	92.66	99.69	92.87	92.7	0.00330
Accuracy (%)	92.96				

**Table 8:** Performance matrices of DenseNet201 model for human action recognition.

Model	Sensitivity	Specificity	Precision	F1-score	FPR
DenseNet201—DenseNet201	92.53	99.69	92.81	92.62	0.00306
Accuracy (%)	92.96				

**Table 9:** Comparison of proposed model with and without feature selection.

Model	Accuracy	Sensitivity	Specificity	Precision	F1-score
Proposed DenseNet model with WCO	0.947	0.9250	0.9920	0.915	0.920
DenseNet model	0.930	0.925	0.997	0.928	0.926

**Table 10:** Comparison of existing works with our model on Standford40 dataset.

Study	Methodology used and results	Merits	Limitations
Proposed Methodology	Various pre-trained models, AlexNet, SqueezeNet, ResNet101 and DenseNet201 were used as feature extractors. Predominant features selected using World cup optimization and predicted using DenseNet classifier. With an accuracy of 94.7%, denseNet201 performed better on Stanford40 dataset.	Double filtering the extracted features using the feature selction process addresses the issues related to overfitting and augmentation.	To improve the action prediction findings the model could be trained on more datasets and using multiple modalities.
Dehkordi et al. (2022)	Super-class learning action recognition (SCLAR) approach + Graph-based class selection (GCS) algorithm + Efficientnet B0 model as classifier. Accuracies on various datasets: Stanford 40 dataset, Pascal VOC 2012 action, BU101 +, IHAR datasets were 92.86%, 92.46%, 92.27% and 92.27% respectively.	Two-phase multi-exprt architecture detect visually distinct differences in the various classes and the framework also overcomes the data imbalace issue.	Domain specific tuning can improve the system performance better.
Dehkordi et al. (2021)	Four lightweight pre-trained CNNs employed for feature extraction. A Feature Attention Module (FAM) selects dominant features before feeding all features to the classifier. They obtained an accuracy of 86.86% on Stanford40 dataset.	Ensemble-based learning avoided the use of any additional annotations which is time consuming and costly.	To get better performance more superior features are required.
Wu & Yu (2020)	Pre-trained VGG-16 with guided Attention Module (GAM) to better locate the discriminative features and a two-level classification network utilising selective search window (SSW). Achieved a result of 87% on PPMI dataset and 89% on Stanford40 dataset.	Part fusion based framework improved the discriminative capability by utilising both image level and part level features.	To improve the action prediction, robust classifier with strong feature identification Technique is required.
Ozcan & Basturk (2020)	Employed NASNet-Large pretrained CNN model and utilised Artificial Bee Colony algorithm (ABC) to finetune the hyperparameters of CNN to achieve optimum result. Obtained an accuracy of 87.78% on Stanford40 dataset.	The İntroduction of an optimized CNN model obtained a better success rate on human action prediction.	Concept of finetuning CNN hyperparameters need to be explored to improve the effectiveness of the model.
Siyal et al. (2020)	Utilised a pre-trained ResNet-18 for feature extraction followed by SVM classifier for action recognition. On Stanford40 dataset, researchers achieved an accuracy of 87.22%.	Incorporating deep feature characteristics, improved the action recognition task performance.	The performance of the model is not consistent for all action categories.
Chan et al. (2019)	Evaluated four pre-trained models ResNet-18, VGG16, VGG19 and googlenet for feature extraction. Principal Component Analysis (PCA) is carried out on extracted features and only principal components are fed to the SVM Classifier. The highest accuracy of 87.13% was achieved by ResNet-18 on Stanford40 dataset.	Using Principal Component analysis reduce computations and attained good performance.	More and better features are required to increase the model performance.

## Conclusion and Future Scope

Recognition of human actions from still images is a major task in scene understanding. In our research work, we have used a transfer learning approach to classify human actions into twenty-four distinct classes by using the Stanford 40 dataset. We have compared the performance of four pre-trained models (AlexNet, SqueezeNet, ResNet101, and DenseNet201) to classify the human action from the still image. We have adopted a feature selection module using the World Cup optimization algorithm to select the most predominant features and improve accuracy. In our proposed model, the features are extracted by the pre-trained DenseNet201 model, and then the World Cup optimizer selects the best features, with the final classification done by the DenseNet classifier. By adding the feature selection module, our proposed model could achieve better performance in analyzing the different human actions in our dataset. Unlike the existing works in this field, no data augmentation or annotation is required, which is time-consuming and costly. An accuracy of 94.7% was obtained with the DenseNet 201 model for human action recognition on our Stanford 40 dataset. In our future work, we will focus on analyzing the performance of multiple modalities using different datasets related to human action recognition to improve the performance of the model.

##  Supplemental Information

10.7717/peerj-cs.1396/supp-1 Supplemental Information 1 Code

## Abbreviations

ABC: Artificial bee colony
AI: Artificial Intelligence
BAO: Bayesian optimization
BoW: Bag-of-words
CNN: Convolutional neural netrork
FC: Fully connected
FIFA: Federation Internationale de Football Association
Fn: False negative
Fp: False positive
FPR: False positive rate
GA: Genetic algorithm
HOG: Histogram oriented gradients
LDA: Linear Discriminant Analysis
LSTM: Long short-term memory
n: Number of members
PCA: Principal component analysis
PSO: Particle swarm optimization
R-CNN: Region based convolution neural network
Resnet: Residual network
SIFT: Scale invarient feature transformation
SUN: Scene UN derstanding
SVM: Support vector machine
Tn: True negative
Tp: True positive
VLAD: Vector of Locally Aggregated Descriptors (VLAD)
WCO: World cup optimization
X: Each continent
*β*: Coefficient
*α*: Standard deviation

## Additional Information and Declarations

### Data Availability

The following information was supplied regarding data availability: The Stanford 40 action dataset is available at: http://vision.stanford.edu/Datasets/40actions.html. The Matlab code is available in the Supplemental File.
